# Combined Effects of Social and Behavioral Factors on Stress and Depression

**DOI:** 10.3390/diseases13020046

**Published:** 2025-02-04

**Authors:** Emmanuel Obeng-Gyasi, Sonya Parker

**Affiliations:** 1 Department of Built Environment, North Carolina A&T State University, Greensboro, NC 27411, USA; 2 Environmental Health and Disease Laboratory, North Carolina A&T State University, Greensboro, NC 27411, USA

**Keywords:** allostatic load, depressive symptoms, social determinants of health, behavioral risk factors, Bayesian Kernel Machine Regression (BKMR)

## Abstract

Background: Chronic stress, driven by the persistent activation of the body’s stress response system—including the sympathetic nervous system and hypothalamic–pituitary–adrenal (HPA) axis—has far-reaching effects on both physical and mental health. This study examines the combined effects of social and behavioral factors on a latent variable consisting of stress and depressive symptoms, using a comprehensive framework to explore the complex interactions of these factors. Methods: Leveraging data from the United States Centers for Disease Control and Prevention’s (CDC’s) National Health and Nutrition Examination Survey (NHANES), we operationalized allostatic load—a measure of cumulative physiological stress—through 10 biomarkers spanning cardiovascular, inflammatory, and metabolic systems. Depressive symptoms were measured via the Patient Health Questionnaire-9 (PHQ-9), and a latent variable capturing the shared variance between stress and depressive symptoms was derived using factor analysis. To assess the influence of social (income and education) and behavioral (alcohol consumption and smoking) factors on this latent variable, we employed Bayesian Kernel Machine Regression (BKMR), allowing us to examine potential non-linear and interactive effects among these predictors. Results: Our results revealed a significant positive association between allostatic load and depressive symptoms across the sample, regardless of ethnic background. Alcohol consumption emerged as a key behavioral factor, with significant positive associations with stress. Conversely, education showed a protective effect, with higher education levels associated with decreased stress and depressive symptoms. Conclusions: These findings underscore the importance of addressing both social determinants and behavioral risk factors in mitigating the cumulative impacts of stress and depressive symptoms. By highlighting the roles of alcohol consumption and education, this study provides insights that can inform public health strategies aimed at promoting resilience and reducing stress-related health disparities.

## 1. Introduction

The body’s stress response system—encompassing the sympathetic nervous system and hypothalamic–pituitary–adrenal (HPA) axis—faces persistent activation in today’s society, leading to chronic stress that affects a broad segment of the population. The cumulative burden, or allostatic load, associated with chronic stress has significant implications for both mental and physical health. Although coping resources and cultural factors can offer resilience, contemporary psychosocial stressors have resulted in widespread and persistent challenges to maintaining biological homeostasis, leading to metabolic and mental health impacts across various demographic groups [1,2,3].

Allostatic load, a measure of cumulative physiological stress, serves as a critical concept in understanding the interplay between chronic stress and health outcomes [4,5]. Rooted in the allostasis framework, which emphasizes the body’s ability to maintain stability through change, allostatic load quantifies the ‘wear and tear’ on biological systems caused by the prolonged activation of stress responses [6,7]. This concept aligns with public health models such as the social determinants of health framework, which highlights the role of socioeconomic and environmental factors in shaping health outcomes [8]. Additionally, the biopsychosocial model underscores how physiological stress interacts with psychological and social domains, providing a comprehensive lens for exploring the multifaceted impacts of allostatic load and mental/physical health outcomes [9].

The HPA axis, crucial in mediating the body’s stress response, releases cortisol, a primary glucocorticoid that helps regulate this response and maintain homeostasis. However, excessive exposure to cortisol due to chronic stress can have detrimental effects, including susceptibility to depressive symptoms [2,3]. Despite increased stress levels across populations, studies indicate that certain groups may experience a higher disease burden not only due to diagnosed depression but also due to underdiagnosed and chronic stress-related conditions [3,10]. In particular, chronic stress is linked to a range of health impacts due to the body’s inability to complete the stress response cycle, which can lead to allostatic load—a state where prolonged stress exposure disrupts physiological balance [10].

Combined social and behavioral factors, including socioeconomic status, employment stability, and social support networks, play a significant role in exacerbating both depression and chronic stress. Social conditions such as limited access to health resources, job insecurity, and social isolation contribute to elevated stress levels and can amplify the effects of allostatic load. Behavioral factors, such as physical inactivity or poor coping mechanisms, further intensify stress response activation and may lead to a higher prevalence of depressive symptoms [11,12]. These combined factors underscore the importance of addressing both social and behavioral dimensions in public health efforts to reduce stress-related health risks and mental health burdens across populations.

Exploring the relationship between depressive symptoms and allostatic load provides insights into how chronic stress exacerbates mental health issues [10,11]. Depression, which includes emotional, cognitive, and physical symptoms, is a leading cause of disability and can severely impair daily functioning and quality of life [12]. The cumulative effects of stress and depression underscore the importance of a mental health model that extends beyond isolated symptoms to include broader physiological responses to chronic stress. Recognizing the physiological wear and tear that results from prolonged exposure to stress hormones is crucial to understanding how depression, as part of mental health disorders, manifests and impacts overall health [4,13].

The Surgeon General’s 2000 report on mental health advocated for integrating mental health into the public health framework, urging the development of a model that addresses both disease and wellness [14]. Depression, identified as a prevalent and costly health condition, was highlighted for its significant impact on public health and its role in exacerbating other health risks.

In public health, attention is increasingly focused on the dual burden of chronic stress and depression and their role in cumulative physiological wear or allostatic load. The chronic activation of the stress response, resulting in persistent cortisol elevation, has been linked to both depressive symptoms and an increased risk of chronic diseases [3]. This relationship underscores the need to address depression as both a consequence and a contributing factor in the context of chronic stress [2].

The physiological and psychological toll of chronic stress, particularly as it relates to depressive symptoms, requires further investigation. Recognizing the disproportionate impact of depression and stress across groups also highlights the need for more inclusive standards of care [15]. By focusing on depressive symptoms within mental health frameworks, public health initiatives can better address the complex relationships between stress, depression, and health disparities. This approach encourages the development of a health system that supports mental well-being by targeting depressive symptoms and chronic stress, which are critical to improving health outcomes and quality of life across diverse populations.

Understanding and measuring the relationship between depressive symptoms and allostatic load is crucial for advancing public health, as it provides valuable insights into mechanisms that, when addressed, may reduce the incidence of chronic disease. This study aims to examine depressive symptoms and chronic stress in U.S. adults, shedding light on social and behavioral factors as predictors of disease risk and mortality to inform strategies that promote psychological and physiological resilience.

## 2. Materials and Methods

### 2.1. Data Collection Procedures

Data for this study were sourced from the Centers for Disease Control and Prevention’s (CDC’s) National Health and Nutrition Examination Survey (NHANES) [16]. NHANES data collection involved initial in-home interviews by trained interviewers to gather demographic, health, and behavioral information. Additional data, including responses to the Patient Health Questionnaire-9 (PHQ-9) for depression screening, were collected at the NHANES Mobile Examination Center (MEC) using a Computer-Assisted Personal Interview (CAPI) system. For this study, deidentified NHANES data were leveraged under secondary data use protocols, ensuring confidentiality. In compliance with section 308(d) of the Public Health Service Act (42 U.S.C. 242 m), participant data confidentiality was assured, negating the need for Institutional Review Board (IRB) oversight.

### 2.2. National Health and Nutrition Examination Survey (NHANES) and Patient Health Questionnaire-9 (PHQ-9)

NHANES, a large-scale survey conducted by the CDC, provides detailed health and nutrition data on a nationally representative sample of U.S. adults. The survey collects a wide range of health and lifestyle information, reflective of diverse U.S. populations. The Patient Health Questionnaire-9 (PHQ-9), a 9-item module within the Patient Health Questionnaire, is a self-administered depression screening tool derived from the PRIME-MD diagnostic framework. It is widely used and validated for identifying major depressive disorder (MDD).

The PHQ-9 scores each symptom on a scale from 0 to 3, based on the frequency of occurrence: 0 (not at all), 1 (several days), 2 (more than half the days), and 3 (nearly every day). Total scores range from 0 to 27 and are categorized to guide clinical response:

- 0–4: None (no action required).
- 5–9: Mild (monitor with a follow-up PHQ-9).
- 10–14: Moderate (treatment plan with potential counseling or medication).
- 15–19: Moderately Severe (active treatment plan with psychotherapy and/or medication).
- 20–27: Severe (immediate pharmacotherapy; if ineffective, referral to a mental health specialist).

This scoring system allows clinicians to assess depression severity and determine an appropriate intervention based on symptom intensity.

### 2.3. Operationalizing Allostatic Load

Informed by previous research [17,18,19,20], we operationalized the allostatic load index using 10 biomarkers spanning cardiovascular, inflammatory, and metabolic systems, based on NHANES data. Cardiovascular biomarkers included systolic blood pressure (SBP), diastolic blood pressure (DBP), triglycerides, HDL cholesterol, and total cholesterol. The inflammatory system was represented by C-reactive protein (CRP), while the metabolic system markers included body mass index (BMI), hemoglobin A1C, albumin, and creatinine clearance. This selection allowed for a comprehensive assessment of cumulative stress effects on physiological systems in the study participants.

### 2.4. Participants

Our study sample consisted of 356 U.S. adults assessed for depression and other health metrics using NHANES data from the 2017–2018 cycle. Inclusion criteria encompassed individuals with complete data on allostatic load, PHQ, alcohol use, smoking status, and education. Exclusion criteria included participants with incomplete or missing data for these variables. The sample was refined using these criteria to ensure robust and reliable analysis. NHANES employs a stratified multi-stage sampling method to ensure representativeness across diverse subpopulations. Participants ranged from 18 to 80 years of age and included individuals from multiple ethnic backgrounds as classified by the CDC: Non-Hispanic White, Non-Hispanic Black, Mexican American, Other Hispanic, Non-Hispanic Asian, and other races, including Multi-racial. This random sampling approach ensured each population group had an equal chance of selection, minimizing overlap.

### 2.5. Data Analysis

We conducted a comprehensive analysis to explore relationships between allostatic load and depressive symptoms (PHQ-9) across ethnic groups. Initially, we computed the mean, standard error (SE), and 95% confidence intervals (CIs) for allostatic load and PHQ-9 scores by ethnicity to summarize central tendencies and variability across groups.

Next, we employed multiple linear regression to examine associations between allostatic load and PHQ-9, incorporating ethnicity as a categorical predictor. This model allowed us to assess both the main effects of allostatic load and ethnicity on depressive symptoms and potential interaction effects, offering insight into whether the relationship between allostatic load and depression varied by ethnicity.

Further, we used factor analysis to identify a latent variable capturing the shared variance between allostatic load and PHQ-9 scores, representing a common dimension related to stress-related health impacts. This latent variable was then analyzed using Bayesian Kernel Machine Regression (BKMR), which allowed us to explore complex, non-linear relationships between exposures and stress-related health outcomes.

BKMR is a Bayesian statistical approach designed to evaluate high-dimensional data, particularly the simultaneous associations of multiple exposures with a health outcome while accounting for potential interactions and non-linear effects. Furthermore, BKMR was utilized to explore the association between exposure mixtures and outcomes based on a kernel function, enabling the modeling of non-linear exposure–response functions and interactive effects between exposures and outcomes [21].

The BKMR model was specified as follows:

Y = h(Z) + βX + ϵ

where Y represents the health outcome, X denotes covariates and their effects, Z indicates the exposure matrix, and h(Z) is the kernel-based function accommodating non-linearity and interactions among exposures. The Gaussian kernel function was used in this study. Models were fitted with 50,000 iterations using a Markov chain Monte Carlo (MCMC) algorithm.

The combined effects of exposure mixtures were estimated by calculating changes in outcome risk at specific percentiles compared to the median percentile. The individual effect of each exposure was assessed by evaluating differences in risk when a single exposure was at the 75th percentile relative to the 25th percentile while holding other exposures fixed at the 25th, 50th, and 75th percentiles. Dose–response relationships and two-way interactions among exposures were visualized using exposure–response curves with other exposures held at specific percentiles.

Unlike Frequentist approaches that rely on *p*-values, BKMR provides posterior distributions for model parameters, allowing credible intervals to quantify uncertainty and directly interpret probabilistic effects. This method enabled us to robustly assess non-linear and interactive relationships while accounting for the complexity of the data.

All analyses were conducted using R (version 4.2.3; R Foundation for Statistical Computing, Vienna, Austria) and Stata SE 15 (StataCorp LLC, College Station, TX, USA). For non-BKMR analyses, a *p*-value of <0.05 was considered statistically significant.

## 3. Results

### 3.1. Sample Characteristics and Descriptive Statistics

The final sample size for this study was 356 individuals. The results of our study revealed that the mean age was 48.83 with a standard deviation of 14.95. The age range was from 20 to 80 years. Table 1 presents the mean allostatic load (AL) and depressive symptom (PHQ) scores, with standard errors (SEs) and 95% confidence intervals (CIs) for each ethnic group. “Other Race—Including Multi-Racial” participants show the highest mean AL at 4.6127, while participants categorized as “Other Race—Including Multi-Racial” also reported the highest mean PHQ score of 6.0197. These descriptive statistics provide an overview of variations in physiological stress and depressive symptoms across ethnic groups.

### 3.2. Correlation Analysis of Key Variables

**Figure 1:**
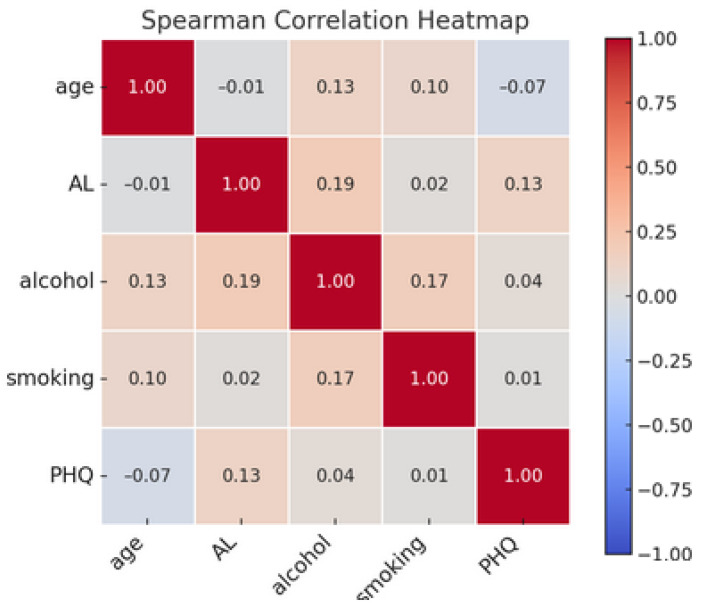
Spearman correlation heatmap of age, allostatic load, alcohol consumption, smoking, and depressive symptoms (PHQ).

Figure 1 presents a Spearman correlation heatmap illustrating the associations among key variables: age, allostatic load, alcohol consumption, smoking, and depressive symptoms (PHQ). The color gradient in the heatmap represents the strength and direction of correlations, with red hues indicating positive correlations and blue hues indicating negative correlations. The color’s intensity reflects the correlation’s magnitude, with darker colors signifying stronger relationships. The diagonal entries, which are all equal to 1.00, denote the perfect correlation of each variable with itself. Off-diagonal elements reveal varying degrees of association between different variables.

### 3.3. Regression Analysis of Allostatic Load and Depressive Symptoms Across Ethnic Groups

Table 2 examines the relationship between allostatic load and depressive symptoms, with ethnicity as a predictor and interaction term. In this analysis, Mexican Americans serve as the reference group, meaning that the coefficients for other ethnic groups (Other Hispanic, Non-Hispanic White, Non-Hispanic Black, Non-Hispanic Asian, and Other Race—Including Multi-Racial) are compared against Mexican Americans as the baseline. Mexican American was chosen as the reference group due to it being a clearly defined and well-characterized group within the U.S. context, which allows for more meaningful comparisons. In contrast, the “Other Race, including Multi-Racial” group encompasses a multitude of diverse racial and ethnic identities, making it less homogenous and harder to interpret or compare systematically.

In the analysis, the main effect of allostatic load was statistically significant, with a coefficient of 1.1214 (*p* = 0.035), indicating a positive association between increased cumulative physiological stress and depressive symptoms. This suggested that, across all groups, individuals with higher allostatic load tended to report more depressive symptoms.

For ethnicity, the coefficients represented the difference in depressive symptoms for each group relative to Mexican Americans, but none were statistically significant. Non-Hispanic White participants had a coefficient of 4.8 (*p* = 0.133), Non-Hispanic Black participants showed a coefficient of 4.0182 (*p* = 0.125), Other Hispanic participants had a coefficient of 1.2791 (*p* = 0.7), Non-Hispanic Asian participants had a coefficient of −1.0839 (*p* = 0.654), and Other Race—Including Multi-Racial participants had a coefficient of 5.2123 (*p* = 0.075). While these coefficients indicated different levels of depressive symptoms compared to Mexican Americans, the lack of statistical significance meant that these differences were not robust when controlling for other variables. The interaction terms between allostatic load and ethnicity also did not yield significant results, indicating that the relationship between allostatic load and depressive symptoms did not significantly differ across ethnic groups in this sample. For instance, the interaction term for Non-Hispanic White participants (Allostatic Load × Non-Hispanic White) had a coefficient of −1.1656 (p = 0.099), the term for Non-Hispanic Black participants (Allostatic Load × Non-Hispanic Black) was −0.1068 (*p* = 0.851), and the term for Non-Hispanic Asian participants (Allostatic Load × Non-Hispanic Asian) was −0.3663 (*p* = 0.463). These non-significant interactions suggested that the impact of physiological stress on depressive symptoms remained consistent across ethnic groups. We also used a reduced linear regression model excluding interaction terms to evaluate the stability of the main effects. The results confirmed that removing interaction terms did not lead to any significant changes in the main effects, and none of the ethnicity categories reached statistical significance. As such, multiple comparison adjustments were not necessary. This highlights the robustness of the observed findings and supports the decision to include interaction terms for a more comprehensive assessment of potential nuanced relationships. Advanced methods like Bayesian Kernel Machine Regression further address complex interactions and non-linear relationships that may not be fully captured by traditional models, underscoring the need for complementary analytic approaches in studies of this nature.

### 3.4. Factor Analysis and Creation of Latent Variable for Combined Stress and Depressive Symptoms

In our study, factor analysis was conducted to investigate the underlying structure of allostatic load and depressive symptoms (Table 3 (a) and (b)). Initially, two factors were extracted, with the first factor accounting for approximately 54.78% of the total variance and the second factor explaining 45.22%. However, since our objective was to create a single latent variable capturing the shared variance between allostatic load and depressive symptoms, only the first factor was retained. This retention was based on the principal component factor method, which identifies the most prominent underlying factor explaining the shared variance between variables.

Both allostatic load and depressive symptoms had equal loadings of 0.7401 on this retained factor, indicating that it effectively represents the variance shared by the two variables. Using this factor structure, we then generated a latent variable, which was calculated via Stata through regression scoring. This approach combined the loadings from allostatic load and depressive symptoms into a single composite score, representing a combined measure of stress-related health impacts.

The resulting latent variable thus provides an integrated score that equally reflects contributions from both allostatic load and depressive symptoms, capturing their shared variance. This composite measure, therefore, serves as a meaningful indicator of the “impact of chronic stress and depression symptoms” or the “burden of combined stress and depression symptoms,” making it suitable for use in further analyses to explore complex associations with other health-related variables.

### 3.5. Bayesian Kernel Machine Regression Analysis of Behavioral and Social Predictors of Combined Stress and Depression

BKMR was applied to analyze the complex, potentially non-linear relationships and interactions among the social and behavioral predictors of combined stress and depression. This approach enabled the simultaneous evaluation of multiple variables, identifying key contributors and their joint effects while accounting for potential non-linear association patterns. The analysis provided insights into how these factors collectively influence stress and depression outcomes.

#### 3.5.1. Hierarchical BKMR Analysis of Behavioral and Social Predictors on Combined Stress and Depression

The BKMR model was structured hierarchically, grouping variables into behavioral (Group 1) and social (Group 2) categories. Posterior inclusion probabilities (PIPs) were generated to assess the relative importance of each predictor in the model, helping to identify which variables contribute the most to explaining the variance in the latent variable representing combined stress and depressive symptoms.

Table 4 shows that within the behavioral category, alcohol has a high conditional PIP (0.9575), indicating a strong association with the latent variable. Within the social category, education also has a relatively high conditional PIP (0.7425), suggesting it plays a more substantial role than income in relation to combined stress and depression.

#### 3.5.2. Univariate Effects of Behavioral and Social Factors on Combined Stress and Depression

**Figure 2:**
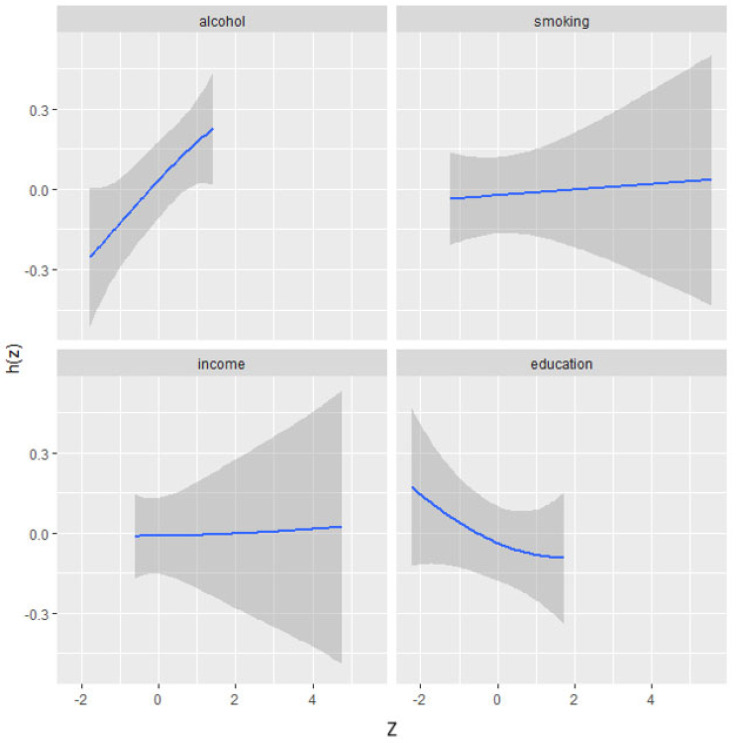
Univariate exposure–response functions and 95% credible interval for association between single factors when other factors are fixed at median. Adjusted for age, ethnicity, and sex. Z: matrix of standardized predictors (e.g., body fat percentage indicators represented as Z-scores). h(Z): kernel-based function in BKMR that models complex, non-linear, and interactive relationships between predictors (Z) and outcome variable.

The univariate analysis visually examines the individual effects of alcohol, smoking, income, and education on combined stress and depressive symptoms. Figure 2 illustrates the impact of each factor on stress/depressive symptoms while holding other factors at their median values and adjusting for covariates. The plot indicates a strong effect of alcohol, showing a positive association with stress and depressive symptoms. 

#### 3.5.3. Bivariate Exposure–Response Analysis of Behavioral and Social Factors of Combined Stress and Depression

**Figure 3:**
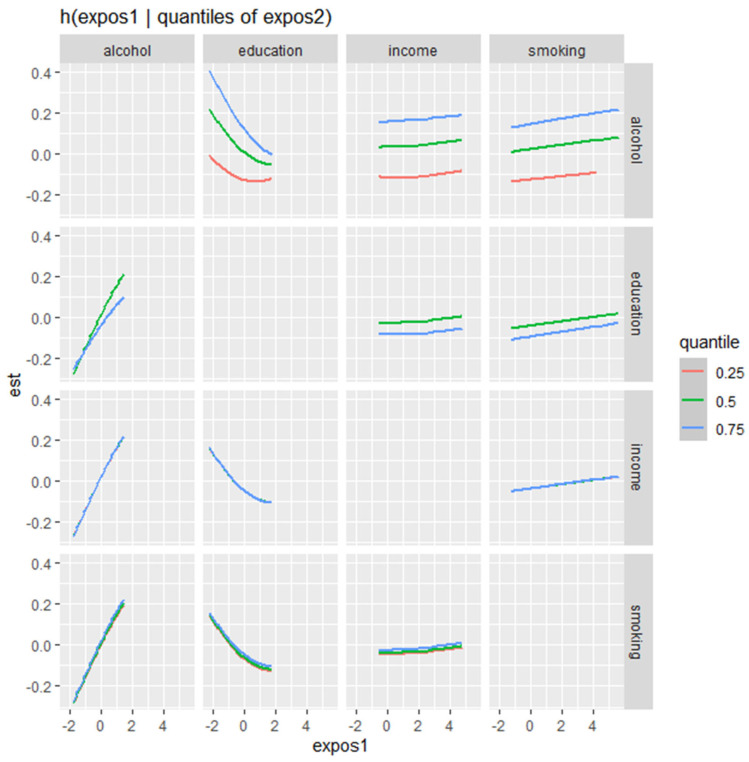
Bivariate exposure–response function of two exposures on combined stress/depressive symptoms—investigating exposure–response function with varying quantiles of second exposure. Adjusted for age, ethnicity, and sex.

Figure 3 presents the bivariate exposure–response functions of various behavioral and social factors (alcohol, education, income, and smoking) of combined stress and depressive symptoms, displaying how the relationship with each predictor changes across different quantiles of a second predictor, while other predictors are held constant.

The plot demonstrates how the effects of various factors on stress and depression outcomes change depending on the level of another factor. Alcohol consumption exhibits a positive association with stress and depression, with a stronger effect observed at higher levels of alcohol intake. This association remains relatively stable across different quantiles of other predictors. Education, on the other hand, shows an inverse relationship with stress and depression symptoms, particularly at higher quantiles, indicating that increased education levels are associated with a reduction in combined stress and depression symptoms. This effect is more pronounced at the 0.75 quantile compared to lower quantiles. Income has a relatively flat effect on stress and depression, suggesting minimal influence overall, though slight variations are noted across different quantiles of other predictors. Smoking, meanwhile, shows a generally positive association with stress and depression symptoms, with a moderate but consistent effect size that varies slightly across different quantiles of other predictors.

#### 3.5.4. Quantile-Based Analysis of Combined Social and Behavioral Factors of Stress and Depressive Symptoms

**Figure 4:**
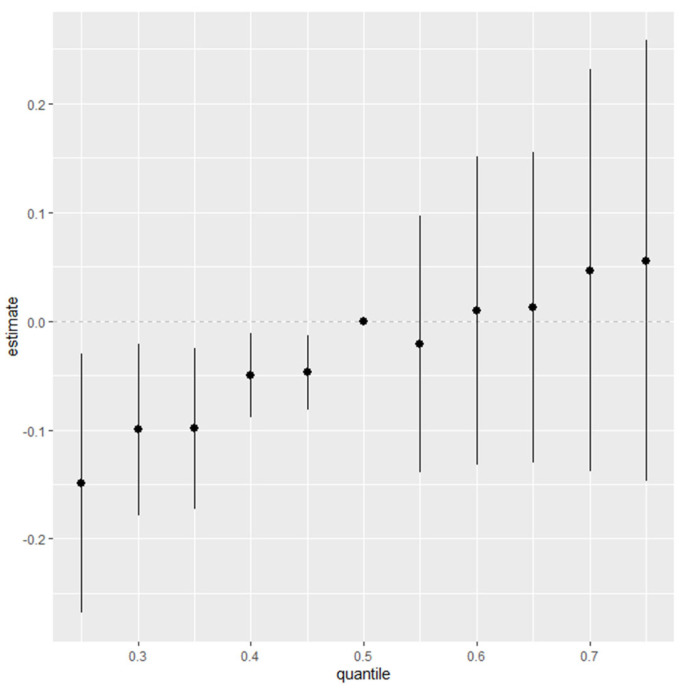
Summary of overall health effects of combined social and environmental factors (alcohol, smoking, income, and education) on stress/depressive symptoms across quantiles (25th to 75th) relative to median. Adjusted for age, ethnicity, and sex. Dots represent estimates, and lines indicate the 95% credible intervals.

Figure 4 presents a quantile-based summary of the effects of combined social and behavioral factors—alcohol consumption, smoking, income, and education—on stress and depressive symptoms. This analysis examines the estimated impact of these factors across various quantiles, from the 25th to the 75th percentile, relative to the median. Each point on the plot represents the estimated effect at a specific quantile, with vertical bars indicating the credible intervals around each estimate.

The results in Figure 4 show a positive trend, indicating that the combined effects of these factors are associated with increased stress and depressive symptoms. This upward trend suggests that as social and behavioral factors collectively intensify, there is a corresponding increase in stress and depressive symptoms, particularly at higher quantiles.

#### 3.5.5. Impact of Individual Variables Within Mixture on Stress and Depressive Symptoms

**Figure 5:**
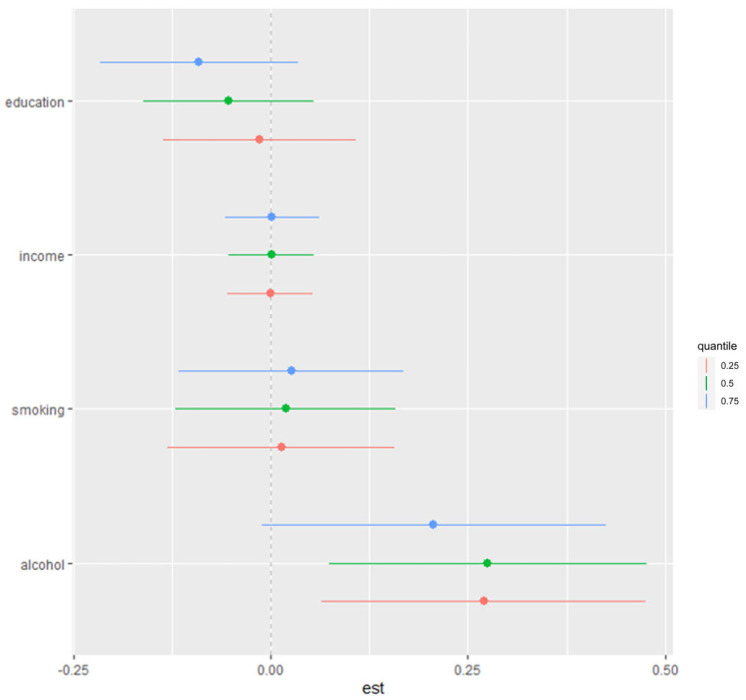
Single-variable association with stress/depressive symptoms. This plot shows change in risks of stress/depressive symptoms with 95% credible interval in single variable, when all other variables were fixed at either 25th, 50th, or 75th percentile. Adjusted for age, ethnicity, and sex. Dots represent estimates, and lines indicate the 95% credible intervals.

We next examined the contribution of individual variables within the mixture to health outcomes by estimating the change in risk for stress and depressive symptoms associated with an interquartile range (IQR) increase in each variable while holding the other variables at the 25th, 50th, or 75th percentiles. The results, shown in Figure 5, indicate that alcohol has a positive and significant effect on stress/depressive symptoms, particularly when other variables (smoking, income, and education) are set at the 25th and 50th percentiles. Specifically, an IQR increase in alcohol consumption is associated with a significant rise in stress and depressive symptoms, suggesting that alcohol is a key factor in this relationship.

Additionally, the effect estimates in Figure 5 suggest potential interactions within the mixture, as the influence of alcohol and other factors varies depending on the percentile level at which the remaining variables are held. This finding underscores the complex, interdependent nature of behavioral and social factors in contributing to stress and depressive symptoms.

## 4. Discussion

### 4.1. Insights into the Interplay of Social and Behavioral Factors of Stress and Depressive Symptoms

The findings of this study offer critical insights into the interplay between social and behavioral factors and their collective influence on stress and depressive symptoms, with significant implications for public health and societal well-being. Leveraging the diversity of the NHANES sample, which includes individuals across various ethnic, socioeconomic, and demographic backgrounds, enhances the generalizability of these findings to the broader U.S. population. This representative sampling approach ensures that key subpopulations, such as Non-Hispanic Black, Non-Hispanic Asian, and Hispanic groups, are adequately included, allowing for a nuanced understanding of how these factors influence stress and depressive symptoms. The analysis highlights the robust relationship between cumulative physiological stress—measured through allostatic load—and depressive symptoms, emphasizing how the chronic activation of the body’s stress response system, when left unresolved, can disrupt mental health [22]. This finding reinforces the concept that allostatic load serves as a tangible indicator of the cumulative burden of stress on the body and its far-reaching effects on psychological well-being. Chronic stress, reflected in heightened allostatic load, contributes to the wear and tear on biological systems, which in turn exacerbates depressive symptoms, creating a vicious cycle of deteriorating health.

The observed racial differences in allostatic load and depressive symptoms did not yield statistically significant results, which may reflect the multifaceted and complex nature of how stress and mental health manifest across different racial groups. While descriptive statistics indicate variations, particularly the high mean allostatic load and PHQ scores among “Other Race—Including Multi-Racial” participants, these differences may not be statistically robust due to sample size limitations or overlapping confidence intervals.

For Non-Hispanic Black participants, the relatively low mean allostatic load and PHQ scores may be partially explained by what some researchers describe as “resilience under stress”. Historical experiences of systemic racism, coupled with strong community ties, cultural coping mechanisms such as spirituality, and the ability to navigate chronic stress, may contribute to adaptive responses that mitigate some of the physiological and psychological effects of stress [23,24]. However, it is important to avoid overgeneralization, as resilience is not uniform and can vary widely within the Black community based on individual circumstances, access to resources, and exposure to structural inequities.

Similarly, the lower PHQ scores observed among Non-Hispanic Asian participants might reflect cultural differences in the expression and reporting of mental health symptoms. Research suggests that individuals from some Asian cultures may internalize mental health struggles or under-report depressive symptoms due to stigma or cultural norms that prioritize emotional restraint [25]. This under-reporting could contribute to the lower mean PHQ scores observed in this group.

The higher mean allostatic load and PHQ scores among “Other Race—Including Multi-Racial” participants could indicate heightened vulnerability due to intersecting stressors. Individuals who identify with multiple racial categories may face unique challenges, such as experiences of identity-related stress or discrimination that do not align with the experiences of mono-racial groups [26,27]. These intersecting stressors could amplify both physiological stress and depressive symptoms.

Additionally, the absence of statistically significant differences across racial groups could stem from the interplay of social determinants of health, which affect all populations to varying degrees. Factors such as socioeconomic status, access to healthcare, neighborhood environments, and educational opportunities are powerful influences that may obscure the effects of race alone when examining stress and depressive symptoms [28,29].

Future research should explore these nuances by incorporating intersectional approaches and considering the cumulative impact of structural inequities and cultural factors. These steps are essential for unraveling the complex interactions that drive disparities in physiological stress and mental health outcomes.

Behavioral factors, particularly alcohol consumption, emerged as powerful contributors to mental health challenges. Alcohol was found to have a substantial and independent association with increased stress and depressive symptoms, with its effects remaining strong even when other factors were controlled. This relationship suggests that alcohol acts as both a symptom and a driver of poor mental health, likely exacerbating stress by disrupting sleep, impairing coping mechanisms, and contributing to neurochemical imbalances [30,31]. Additionally, alcohol consumption has been shown to exacerbate physiological stress responses through mechanisms such as the activation of the hypothalamic–pituitary–adrenal axis, leading to increased cortisol production and prolonged stress arousal. This heightened physiological response not only amplifies stress but also impairs recovery, further perpetuating the cycle of poor mental health. The consistent impact of alcohol across varying quantiles of other predictors further underscores its pervasive role in mental health outcomes. This finding aligns with prior research documenting alcohol’s detrimental effects on mental health but also adds nuance by exploring its interaction with broader social and behavioral contexts.

Conversely, education was identified as a critical protective factor, with higher levels of education associated with significant reductions in stress and depressive symptoms. This protective effect likely stems from several mechanisms: education increases access to resources, improves problem-solving skills, and facilitates healthier behaviors, all of which help mitigate the impact of stressors [32,33]. Moreover, education fosters social mobility and empowerment, enabling individuals to navigate challenges more effectively. These findings align with longstanding evidence in the social determinants of health literature, which highlights education as a key driver of mental and physical health resilience [34]. However, the strength of education’s protective effects in this study, particularly in reducing the shared burden of stress and depressive symptoms, underscores its importance as a public health priority.

The implications of these findings are profound. From a behavioral perspective, this study highlights the urgent need for targeted interventions to address alcohol consumption as a key modifiable risk factor for mental health issues. Public health strategies could include community-based alcohol reduction programs, public awareness campaigns about alcohol’s effects on mental health, and integrated approaches that combine mental health counseling with substance use support. From a social perspective, this study underscores the transformative potential of education as a tool for promoting mental resilience and reducing health disparities. Policies aimed at expanding access to quality education, particularly in underserved communities, could have far-reaching effects not only on individual mental health but also on broader social equity [33,35].

This study also provides a critical framework for understanding the complex interplay between stress, depression, and the social and behavioral determinants of health. Unlike approaches that examine these factors in isolation, this research integrates them into a unified model, revealing the interdependent and synergistic effects they exert on mental health. For instance, the interaction between alcohol consumption and socioeconomic variables like education and income highlights the compounded risks faced by individuals who experience both behavioral vulnerabilities and limited social resources. These insights underscore the need for public health interventions that address the root causes of stress and depression through a multi-dimensional approach [36,37,38].

Furthermore, while education was shown to have a protective effect, this relationship may be moderated by other unmeasured social determinants, such as neighborhood environments, access to healthcare, or the availability of social support networks [39]. Education likely mitigates stress and its effects on mental health through several mechanisms. For instance, higher education levels may enhance individuals’ access to resources, such as stable employment and healthcare, which can buffer against stress. Additionally, education may foster the development of adaptive coping strategies, problem-solving skills, and a stronger sense of control, all of which are critical for managing stress and reducing its impact on mental health. Future studies should explore these dimensions to develop a more comprehensive understanding of how education interacts with other factors to influence mental health. Intervention-based research is also needed to test the efficacy of programs that simultaneously target alcohol use and promote educational opportunities in mitigating stress and depression.

This study found no statistically significant differences in depressive symptoms across ethnic backgrounds when compared to Mexican Americans as the reference group, despite a significant positive association between allostatic load and depressive symptoms (coefficient = 1.1214; *p* = 0.035). This indicates that higher cumulative physiological stress is linked to greater depressive symptoms across all groups, but the specific burden of depressive symptoms does not significantly vary by ethnicity in this sample.

While ethnic groups showed different coefficients relative to Mexican Americans, these differences were not statistically significant: Non-Hispanic White (4.8, *p* = 0.133), Non-Hispanic Black (4.0182, *p* = 0.125), Other Hispanic (1.2791, *p* = 0.7), Non-Hispanic Asian (−1.0839, *p* = 0.654), and Other Race—Including Multi-Racial (5.2123, *p* = 0.075). The lack of significant differences suggests that depressive symptoms may not be strongly influenced by ethnicity alone when other factors are controlled for.

Similarly, interaction terms between allostatic load and ethnicity were non-significant, indicating that the impact of physiological stress on depressive symptoms does not vary substantially across groups. This consistency across ethnicities may reflect the universal physiological effects of stress, though cultural, systemic, and diagnostic factors could influence how depressive symptoms are experienced or reported within these groups.

### 4.2. Limitations

Despite its strengths, this study is not without limitations. The cross-sectional design precludes causal inferences, leaving open questions about the directionality of observed relationships. For example, while alcohol consumption exacerbates stress and depressive symptoms, it is also possible that individuals with pre-existing mental health challenges are more likely to engage in heavy drinking as a coping mechanism [40,41]. The reliance on self-reported data for alcohol and smoking behaviors introduces potential biases, such as recall bias or social desirability bias, which may lead participants to under-report behaviors deemed socially undesirable. This could result in an underestimation of the true associations between these behaviors and the observed outcomes. Additionally, self-reported measures are subject to individual interpretation, which may introduce variability and affect the reliability of the data. Future research should employ longitudinal designs to clarify the temporal relationships between stress, depressive symptoms, and their predictors and consider incorporating objective measures, where possible, to mitigate the potential biases associated with self-reported data.

## 5. Conclusions

In conclusion, this study offers a nuanced understanding of the social and behavioral predictors of stress and depressive symptoms, emphasizing the dual importance of reducing harmful behaviors like alcohol consumption and strengthening protective factors like education. By addressing the root causes of stress and depression through targeted public health strategies, it is possible to alleviate the mental health burden on individuals and society. These findings call for a renewed focus on integrative approaches that address both individual behaviors and structural inequalities, paving the way for a healthier, more resilient population.

## Figures and Tables

**Table 1:** Mean allostatic load and PHQ scores by ethnicity.

Ethnicity	Mean Allostatic Load	SE Allostatic Load	CI Allostatic Load	Mean PHQ	SE PHQ	CI PHQ
Mexican American	4.2296	0.2862	3.6196, 4.8396	5.0682	0.7371	3.4972, 6.6391
Other Hispanic	3.6062	0.5801	2.3687, 4.8438	5.876	1.2775	3.1532, 8.5988
Non-Hispanic White	3.5735	0.1775	3.1952, 3.9518	4.967	0.5938	3.7014, 6.2325
Non-Hispanic Black	2.9964	0.2148	2.5387, 3.4542	4.4955	0.5053	3.4186, 5.5724
Non-Hispanic Asian	3.3493	0.3598	2.5824, 4.1163	1.7706	0.4471	0.8177, 2.7235
Other Race—Including Multi-Racial	4.6127	0.4111	3.7366, 5.4889	6.0197	0.9326	4.0319, 8.0074

CI—confidence interval.

**Table 2:** Regression results for PHQ in terms of allostatic load and ethnicity interactions.

Variable	Coefficient	Std. Err.	*p*-Value	95% Conf. Interval
Allostatic Load	1.1214	0.4831	0.035	0.0916, 2.1512
Other Hispanic	1.2791	3.2537	0.7	−5.656, 8.2142
Non-Hispanic White	4.8	3.0224	0.133	−1.6421, 11.242
Non-Hispanic Black	4.0182	2.4749	0.125	−1.257, 9.2934
Non-Hispanic Asian	−1.0839	2.3677	0.654	−6.1306, 3.9628
Other Race—Including Multi-Racial	5.2123	2.726	0.075	−0.5981, 11.0226
Allostatic Load × Other Hispanic	0.0631	0.7564	0.935	−1.5492, 1.6755
Allostatic Load × Non-Hispanic White	−1.1656	0.655	0.099	−2.5617, 0.2304
Allostatic Load × Non-Hispanic Black	−0.1068	0.5641	0.851	−1.2072, 0.9937
Allostatic Load × Non-Hispanic Asian	−0.3663	0.4949	0.463	−1.4212, 0.6887
Allostatic Load × Other Race—Including Multi-Racial	−1.0168	0.6237	0.119	−2.3462, 0.3125

**Table 3:** (a) Factor analysis results for allostatic load and PHQ factor summary. (b) Factor loadings and uniqueness.

(a)						
**Factor**	**Eigenvalue**		**Difference**	**Proportion**		**Cumulative**
Factor1	1.0956		0.1912	0.5478		0.5478
Factor2	0.9044			0.4522		1.0
(b)						
**Variable**		**Factor1 Loading**			**Uniqueness**	
Allostatic Load		0.7401			0.4522	
PHQ		0.7401			0.4522	

**Table 4:** Posterior inclusion probabilities (PIPs) for behavioral and social predictors of combined stress and depression.

Variable	Group	groupPIP	condPIP
alcohol	1	0.8992	0.9575
smoking	1	0.8992	0.0425
income	2	0.5118	0.2575
education	2	0.5118	0.7425

## Data Availability

The data presented in this study are openly available on the CDC NHANES site at https://wwwn.cdc.gov/nchs/nhanes/continuousnhanes/overview.aspx?BeginYear=2017 (accessed on 1 December 2024).
